# Not there yet: using data-driven methods to predict who becomes costly among low-cost patients with type 2 diabetes

**DOI:** 10.1186/s12902-020-00609-1

**Published:** 2020-08-17

**Authors:** Julie C. Lauffenburger, Mufaddal Mahesri, Niteesh K. Choudhry

**Affiliations:** 1 Center for Healthcare Delivery Sciences (C4HDS), Department of Medicine, Brigham and Women’s Hospital and Harvard Medical School, 1620 Tremont Street, Suite 3030, Boston, MA 02120 USA; 2 Division of Pharmacoepidemiology and Pharmacoeconomics, Department of Medicine, Brigham and Women’s Hospital and Harvard Medical School, 1620 Tremont Street, Suite 3030, Boston, MA 02120 USA

**Keywords:** Diabetes, Costs of care/healthcare expenditures, Healthcare management, Medicare

## Abstract

**Background:**

Diabetes is a leading cause of Medicare spending; predicting which individuals are likely to be costly is essential for targeting interventions. Current approaches generally focus on composite measures, short time-horizons, or patients who are already high utilizers, whose costs may be harder to modify. Thus, we used data-driven methods to classify unique clusters in Medicare claims who were initially low utilizers by their diabetes spending patterns in subsequent years and used machine learning to predict these patterns.

**Methods:**

We identified beneficiaries with type 2 diabetes whose spending was in the bottom 90% of diabetes care spending in a one-year baseline period in Medicare fee-for-service data.

We used group-based trajectory modeling to classify unique clusters of patients by diabetes-related spending patterns over a two-year follow-up. Prediction models were estimated with generalized boosted regression, a machine learning method, using sets of all baseline predictors, diabetes predictors, and predictors that are potentially-modifiable through interventions. Each model was evaluated through C-statistics and 5-fold cross-validation.

**Results:**

Among 33,789 beneficiaries (baseline median diabetes spending: $4153), we identified 5 distinct spending patterns that could largely be predicted; of these, 68.1% of patients had consistent spending, 25.3% had spending that rose quickly, and 6.6% of patients had spending that rose progressively. The ability to predict these groups was moderate (validated C-statistics: 0.63 to 0.87). The most influential factors for those with progressively rising spending were age, generosity of coverage, prior spending, and medication adherence.

**Conclusions:**

Patients with type 2 diabetes who were initially low spenders exhibit distinct subsequent long-term patterns of diabetes spending; membership in these patterns can be largely predicted with data-driven methods. These findings as well as applications of the overall approach could potentially inform the design and timing of diabetes or cost-containment interventions, such as medication adherence or interventions that enhance access to care, among patients with type 2 diabetes.

## Background

Type 2 diabetes is one of the most prevalent and most costly conditions for Medicare; in 2017, more than 200 billion US dollars was spent on direct costs related to diabetes, of which 61% was by older adults ≥65 years of age [1, 2]. Fortunately, the burden of type 2 diabetes can be substantially reduced through medication and lifestyle interventions [3–5]. Despite this, patients living with type 2 diabetes frequently develop kidney disease, another significant driver of healthcare costs [3]. These types of diabetes-related complications are some of the key contributors to rising costs in these patients [3].

There are several possible explanations for the limited success in mitigating these rising costs. First, interventions for type 2 diabetes often focus on patients who have already become costly or poorly controlled, even though these patients may only represent a fraction of those who could benefit from an intervention [1, 2, 6]. Second, while accurately predicting which individuals are likely to become high cost is essential for targeting interventions [7–12], current approaches to predicting spending generally focus on composite measures (like mean costs) and short time horizons, even though patients with the same condition may have costs and seek care in ways that fluctuate over time [13, 14]. For example, patients with type 2 diabetes who are hospitalized early in a calendar year may differ meaningfully from those who are hospitalized later in the year in terms of how that patient should be managed, although composite metrics would classify them similarly [15, 16].

Research in other settings has observed similar healthcare cost dynamics. For instance, Tamang et al. identified a definable group of low-spending patients whose costs “bloomed”, or became costly, in the following year within the general Danish population [17]. Similarly, Lauffenburger et al. observed seven dynamic patterns of spending among a large sample of US commercially-insured beneficiaries, including individuals whose costs increased rapidly towards the end of the year and another group of relatively high cost individuals for whom spending fell [18].

These approaches have not yet been applied to specific spending among patients with a chronic disease, such as type 2 diabetes. In specific, little is known about the patterns of spending among patients with type 2 diabetes who are currently low utilizers and how many and when these patients may become costly to Medicare, the US national health insurance program for many US older adults. The ability to better proactively discriminate between patients with diabetes who have increasing spending over time could better target interventions to those at greatest need. Therefore, we used a dynamic, data-driven approach to classify individuals with diabetes by their long-term diabetes-specific spending patterns and assessed the ability to predict membership in these groups.

## Methods

### Setting and study design

We used data from a 1-million-member sample of Medicare Fee-for-Service beneficiaries, including Medicare Parts A (inpatient services), B (outpatient services) and D (prescription drugs) patient-level files, from 2011 to 2013; this original sample included ~ 20,000 beneficiaries in a nationwide quality improvement program and ~ 980,000 randomly selected patients nationally [19]. We restricted the cohort to the randomly-selected patients. Medicare Fee-for-Service plans comprise the vast majority of insurance coverage options for US older adults ≥65 years of age; Fee-for-service in particular means that services, such as hospitalizations, medications, and procedures are paid for individually. Thus, these data contain complete paid administrative claims for all procedures, physician encounters, hospitalizations, and outpatient prescription dispensations, including amounts paid by the insurer and the patient, linked to eligibility data. Aggregate data on socioeconomic status were obtained by linking each beneficiary’s zip code of residence with 2010 United States Census data. The Brigham and Women’s Hospital Institutional Review Board approved the study.

To be included in the cohort, patients had to be ≥65 years of age, have a validated diagnosis of type 2 diabetes (i.e., two outpatient or one inpatient diagnoses) [6, 20] in the baseline year, and maintain continuous eligibility in Medicare Parts A, B, and D from January 1, 2011 to December 31, 2013. The entry date for the cohort was defined as January 1, 2012. The baseline year spanned January 1, 2011 to December 31, 2011 (Appendix Figure 1). We further restricted the study cohort to those who previously had lower spending levels (hereafter referred to as “low-cost”), defined as being in the 90% of spending in the baseline year (see “Costs” for further details) [17].

### Costs

We measured total monthly healthcare spending for each eligible patient for care related to diabetes by summing each individual’s allowed costs for inpatient visits, outpatient medical and physician office visits, and outpatient medications beginning with the entry date for the cohort [6]. We focused on diabetes costs in particular because they may be more modifiable than other types of spending by patients. To define costs for care related to diabetes, diabetes costs for inpatient visits were identified by searching for hospitalizations where type 2 diabetes was recorded as the primary diagnosis (i.e., International Classification of Diseases 9th edition (ICD-9) codes 250.×0 and 250.×2) [6, 19]. As in prior validation work, outpatient medical and physician office visits were identified by searching for medical claims with any diagnosis of type 2 diabetes.^6.19^ We also measured the use and associated costs of all diabetes medications for which an outpatient claim was generated (Appendix Table 1). Monthly costs were calculated by adding up these costs per month, dividing by the number of days in that month, and multiplying by 30 to generate standardized values. These costs were then logarithmically transformed to normalize their distribution, as is frequently done in healthcare spending [12, 21]. Costs were inflated using the consumer price index medical care component to 2013 costs, as necessary.

As in prior research, we defined patients as low-cost if they were in the lower 90% of overall diabetes spending in the baseline year [17]. The appropriateness of this threshold in the baseline year was confirmed using percentiles within the Medicare data (Appendix Figure 2) but was explored in sensitivity analyses using a lower threshold (i.e., 40%).

### Baseline predictors

Sociodemographic predictors included age, race/ethnicity and gender directly from the enrollment files as well as zip-code level variables, such as median household income and educational attainment.

We specified clinically-relevant characteristics using data in the baseline year. These variables were based on characteristics that have been used in cost-modeling in administrative claims data as well as from the Quality-Cost theoretical framework [9, 17, 18, 22]. These predictors have also been shown in prior work to be as predictive as proprietary risk-adjustment methods [18]. Clinical factors were measured using ICD-9 codes and included comorbidities such as coronary artery disease, prior myocardial infarction, hypertension, congestive heart failure, stroke, major depression, liver disease, chronic kidney disease, atrial fibrillation, Alzheimer’s/dementia, osteoporosis, obesity, and tobacco use.

We measured each beneficiaries’ numbers of unique medications (by generic name), emergency room visits, outpatient physician office visits, hospitalizations, unique physicians, and unique pharmacies used during the baseline period. We also measured adherence to common chronic medication classes, including diabetes medications [18]. For this calculation, for each therapeutic class (such as beta-blockers), we created a “supply diary” beginning with the first fill for each medication in the first 6 months of the baseline year and linked all subsequent observed fills based on dispensing date and days’ supply; switching was allowed within each therapeutic class. From this diary, we calculated the proportion of days covered (PDC) as an average across any class that the patient filled to generate one mean PDC [23, 24].

We measured diabetes-specific predictors, including use of insulin, non-insulin injectables (e.g., GLP-1 agents), number of oral glucose-lowering agents, use of testing supplies (listed in Appendix Table 1). In addition, we measured adherence to oral glucose-lowering agents for type 2 diabetes (e.g., by calculated an average PDC using the same method as above) and persistence to insulin (defined as having < 90 day gap in supplies based on the supply diaries) [25].

Finally, we classified whether each of these baseline predictors were potentially-modifiable, defined by whether they could theoretically be addressed in an intervention and by categorizations in other research [26, 27]. For example, the number of unique physicians that a patient sees could be potentially-modifiable, while patient’s race/ethnicity status would not be. On this basis and using classifications for potentially-modifiable based on prior work, we classified 10 of the baseline predictors as potentially-modifiable [25, 26].

### Data-driven approach to modeling diabetes costs

We used trajectory modeling to empirically classify spending patterns during the two-year follow-up period. This approach considers changes in healthcare spending over time, rather than aggregating costs over a set time period [28]. Group-based trajectory models are an application of finite mixture modeling that identify clusters of individuals who follow similar patterns over time [29]. This approach fits a semiparametric (discrete) mixture model to longitudinal data. Using this method, we modeled longitudinal cost trajectories in the two-year follow-up period using calendar month as the time variable, diabetes-related costs in each of those 24 months, a censored normal distribution, and a third-order polynomial [18, 29, 30]. On the basis of these models, we used the probability of membership in each group for each individual to assign patients to the trajectory group with the highest membership probability, as in prior approaches [28].

We estimated each of these trajectory models using a “forward” classifying approach from 2 to 6 groups, each time investigating model fit using the Bayesian information criterion (BIC), in which lower BICs indicate better model fit [29]. The number of groups that we investigated was capped at 6 based on the trajectory groupings observed in prior work [18, 28]. As recommended in the literature, along with considering BIC, we selected the best fitting trajectory model based on the ability to interpret separate groups visually, minimum membership probabilities in each group, and having ≥5% of the sample in each group [30–32]. We used the SAS procedure Proc Traj to implement our analysis [28–30].

### Statistical analysis

Once we selected the best-fitting trajectory model, we assessed the ability to predict membership in each two-year trajectory group using generalized boosted regression models. The boosted algorithm is a non-parametric machine learning method and is thought to be one of the best approaches for prediction [33, 34]. In brief, the algorithm builds numerous small regression trees that together provide highly-accurate classification within a prediction model [35]. The algorithm also automatically selects variables, protects from model overfitting, and describes the relative influence of each predictor [36]. We chose to use this approach rather than fitting covariates in the trajectory models themselves as the boosted algorithm uses these automatic methods to improve model selection.

We estimated three separate boosted regression models for predicting each trajectory group compared with the other trajectory groups. The first model included all baseline predictors (Model 1). The second model included only diabetes-specific predictors (Model 2, predictors shown in Table 1). The third model included only the potentially-modifiable predictors (Model 3, predictors shown in Table 1). To generate the boosted regression models and avoid over-optimism bias, we used the gbm package in R with 5-fold cross-validation and applied standard default values for tuning parameters to identify the optimal model [33]. We evaluated each of these models through discrimination measures, or the ability of the model to distinguish between patients who do and do not experience the outcome [37]. In specific, discrimination was measured by the C-statistic, which ranges from 0.5 (non-informative model) to 1.0 (perfect prediction) [38, 39]. For clinical context, we also explored the relative influence of each predictor from the boosted regression models for Models 2 and 3 to provide insight into baseline factors that may help distinguish patients who may become costly later, such as for example, tobacco use.

**Table 1:** Patient characteristics by diabetes spending trajectory

BASELINE YEAR COVARIATES	Group 1: Minimal user (***N*** = 4907)	Group 2: Low cost (***N*** = 8.525)	Group 3: Rising cost (***N*** = 2215)	Group 4: Moderate cost (***N*** = 9594)	Group 5: High cost (***N*** = 8548)
**Demographics**					
Age, mean (SD)	76.3 (7.0)	75.6 (6.6)	75.9 (6.5)	75.9 (6.6)	75.9 (6.7)
Female sex, %	52.7	51.5	51.4	57.4	61.4
Race/ethnicity, %					
Non-Hispanic White	85.6	85.4	83.8	82.4	78.3
Black	8.3	8.4	10.0	11.4	14.0
Other	2.8	3.3	2.6	2.4	2.5
Asian/Pacific Islander	1.7	1.4	1.5	1.7	2.0
Hispanic	1.7	1.5	2.2	2.2	3.2
Zip code median income, mean (SD)	$52,822 (22,467)	$52,258 (20,643)	$52,568 (19,731)	$51,361 (20,502)	$49,546 (20,657)
Zip code % high school grad, mean (SD)	82.9 (17.3)	83.4 (16.6)	83.8 (14.8)	82.6 (16.4)	81.0 (16.8)
**Healthcare utilization**					
Part D low income subsidy, %	15.1	12.0	13.0	22.7	43.8
No. of office visits, mean (SD) ^a^	8.5 (7.3)	8.9 (6.8)	10.0 (7.5)	10.7 (7.9)	12.1 (9.5)
No. of physicians, mean (SD) ^a^	2.1 (1.2)	1.9 (1.0)	2.0 (1.1)	2.0 (1.1)	2.3 (1.2)
No. of pharmacies used, mean (SD) ^a^	0.8 (1.2)	0.7 (1.1)	0.7 (1.1)	1.2 (1.3)	1.5 (1.2)
No. of hospitalizations, mean (SD) ^a^	0.7 (1.0)	0.4 (0.7)	0.4 (0.7)	0.4 (0.7)	0.5 (0.8)
No. of ER visits, mean (SD) ^a^	0.8 (1.5)	0.6 (1.0)	0.7 (1.1)	0.7 (1.5)	1.0 (1.5)
No. of unique drugs, mean (SD) ^a^	4.4 (6.5)	4.0 (6.0)	4.1 (6.4)	7.5 (7.1)	12.9 (8.1)
Prescription generosity, mean (SD)	0.1 (0.2)	0.1 (0.2)	0.1 (0.2)	0.2 (0.2)	0.2 (0.2)
Medical benefits’ generosity, mean (SD)	0.1 (0.1)	0.1 (0.1)	0.1 (0.1)	0.1 (0.1)	0.1 (0.1)
Total baseline year costs, mean (SD)	$20,835 (28.893)	$14,191 (17,350)	$16,085 (17,205)	$17,353 (17,885)	$24,557 (21,441)
Chronic medication use, %	38.9	37.1	35.2	62.8	83.3
Average adherence, mean (SD) ^a^	0.8 (0.2)	0.8 (0.2)	0.8 (0.2)	0.8 (0.2)	0.8 (0.2)
**Diabetes-specific**					
No. of oral diabetes drugs, mean (SD)	0.1 (0.4)	0.3 (0.6)	0.4 (0.8)	1.0 (1.0)	1.2 (1.1)
Diabetes average adherence, mean (SD)	0.6 (0.3)	0.8 (0.2)	0.8 (0.2)	0.9 (0.2)	0.9 (0.2)
Insulin use, %	0.8	2.2	4.9	11.6	40.4
Insulin persistence, %	36.8	61.1	62.4	77.5	87.1
Hypoglycemia, %	1.4	1.8	1.9	2.9	5.5
Ketoacidosis, %	0.6	0.6	0.9	1.2	2.0
Retinopathy, %	2.8	7.5	11.3	12.7	20.2
Nephropathy, %	0.6	1.2	1.3	2.2	3.6
Neuropathy, %	6.1	13.8	18.2	25.4	36.1
No. of testing supply fills, mean (SD)	0.1 (0.2)	0.1 (0.4)	0.1 (0.7)	0.2 (0.9)	0.7 (2.2)
Baseline year diabetes costs, mean (SD)	$5868 (7476)	$5644 (7313)	$6535 (7386)	$7147 (7437)	$9321 (8902)
**Comorbidities**					
Comorbidity score, mean (SD)	2.1 (3.0)	1.7 (2.5)	2.1 (2.6)	2.3 (2.7)	2.4 (2.7)
Coronary artery disease, %	18.5	12.4	15.3	14.6	19.0
Prior MI, %	2.5	1.3	1.0	1.2	1.4
Asthma or COPD, %	28.8	23.7	25.3	26.3	33.7
Hypertension, %	91.2	92.6	93.1	94.5	96.4
Renal failure or ESRD, %	9.4	5.9	7.7	7.8	12.9
Dementia, %	5.5	2.9	4.1	3.7	7.0
Depression, %^a^	14.5	11.1	13.8	13.3	19.6
Stroke, %	3.1	1.8	2.0	2.1	2.5
Liver disease, %	1.0	0.7	0.8	0.8	1.0
Congestive heart failure, %	8.8	5.3	5.5	6.7	11.2
Hyperlipidemia, %	81.4	87.0	86.7	88.7	87.6
Atrial fibrillation, %	9.8	5.5	6.0	6.5	7.5
Osteoporosis, %	20.6	17.8	18.4	20.4	22.2
Obesity, %^a^	12.8	13.9	16.8	16.0	22.0
Acute stress, % ^a^	6.5	3.7	4.3	4.3	6.6
Tobacco use, % ^a^	18.5	14.5	15.4	15.0	15.4

*Abbreviations*: *SD* Standard Deviation, *COPD* Chronic Obstructive Pulmonary Disease, *ER* Emergency Room, *MI* Myocardial infarction

^a^Potentially-modifiable predictors

In sensitivity analyses, we restricted the cohort as well as subsequent trajectory and prediction modeling to those in the bottom 40% of spending in the baseline year [26]. All analyses except for the boosted regression were performed using SAS 9.4 (Cary, NC). The boosting algorithm was performed in R, Version 3.4.1. This study follows the Strengthening the Reporting of Observational Studies in Epidemiology (STROBE) reporting guidelines.

## Results

### Cohort characteristics

After applying eligibility criteria, our study cohort consisted of 33,789 Medicare beneficiaries with type 2 diabetes were in the bottom 90% of spending at baseline (Appendix Table 2). Their mean age was 75.9 years (Standard Deviation [SD]: 6.7), and 55.8% were female. Median diabetes spending in the baseline year was $4153.

### Trajectory models of diabetes spending

**Fig. 1:**
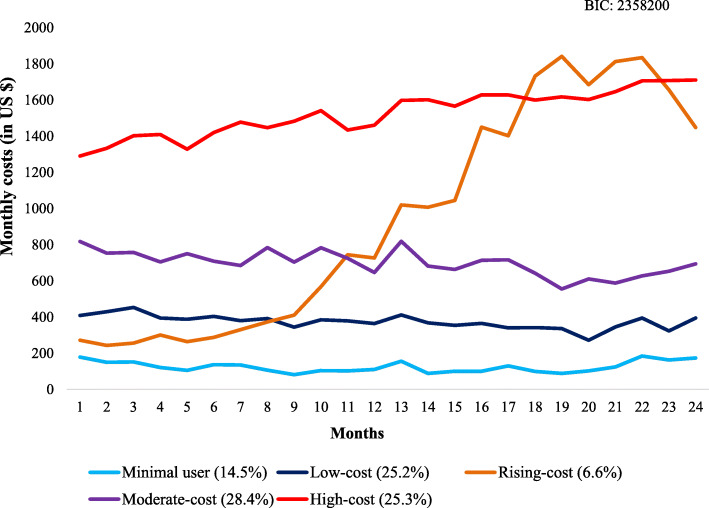
Two-year diabetes spending patterns using trajectory modeling

A 5-group trajectory model best described the two-year diabetes spending patterns (Fig. 1). This final 5-group model included a minimal-user group (Group 1: 14.5%), a low-cost group (Group 2: 25.2%), a rising-cost group (Group 3: 6.6%) whose costs began to rise progressively in the first year of follow-up, a moderate-cost group (Group 4: 28.4%), and a high-cost group (Group 5: 25.3%). Trajectory modeling approaches using other numbers of groups and their corresponding BICs are shown in Appendix Figure 3; other fit criteria for these trajectories are shown in Appendix Table 3. Other models did not meet the best-fitting criteria, including BIC.

Baseline characteristics for each trajectory group are shown in Table 1 (asterisks are shown for the 10 potentially-modifiable factors). Patients across all 5 groups were similar in age. Their baseline spending patterns (in the prior year) by trajectory group are shown in Appendix Figure 4; of note, those in the high-cost trajectory group had slightly higher costs than other trajectory groups, but other groups were largely similar.

### Prediction of diabetes spending trajectories

The cross-validated results of the three main prediction models are shown in Table 2. Most of the two-year diabetes spending trajectory groups could be accurately predicted using all baseline predictors (Model 1), particularly the minimal-user (C-statistic: 0.874), low-cost (C-statistic: 0.746), and high-cost groups (C-statistic: 0.872). The ability to predict the rising-cost (C-statistic: 0.650) and moderate-cost groups (C-statistic: 0.685) was modest. Using diabetes predictors alone (Model 2), overall predictive ability remained modest to strong, for example, the high-cost group had a C-statistic of 0.855. Predictive ability was slightly lower but still relatively similar to the full baseline model (Model 1) using the potentially-modifiable predictors alone (Model 3).

**Table 2:** Model discriminative ability to predict two-year diabetes spending trajectory groups

	Validated C-statistics		
Group (Ref: other groups)	Model 1: All baseline predictors	Model 2: Diabetes predictors	Model 3: Potentially-modifiable predictors
Group 1: Minimal user	0.874	0.847	0.820
Group 2: Low cost	0.746	0.731	0.712
Group 3: Rising cost	0.650	0.632	0.625
Group 4: Moderate cost	0.685	0.675	0.646
Group 5: High cost	0.872	0.855	0.835

**Fig. 2:**
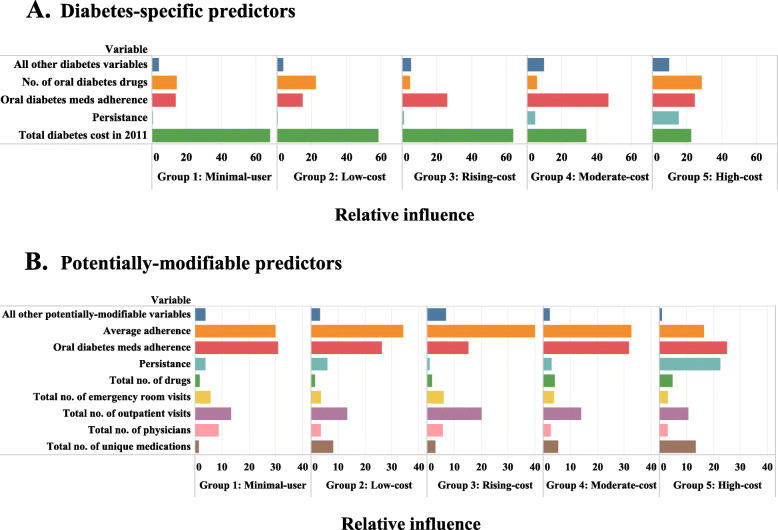
Relative influence of variables for predicting group membership for models including diabetes-specific and potentially-modifiable predictors

The relative influences of predictors from the boosted regression for Model 2 (Diabetes-specific) and Model 3 (Potentially-modifiable predictors) for each of the 5 trajectory groups are shown in Fig. 2 (collapsed across predictors with relative influence < 5) and Appendix Figure 5 (uncollapsed). In brief, the most influential predictors depended on the model and group being predicted. For example, the most influential factor for Groups 1–3 in Model 3 were baseline diabetes spending, while for Group 4, the most influential factor was average adherence to oral diabetes medications and for Group 5, it was number of unique diabetes medications. When examining the group with progressively rising spending (Group 3) in particular, in Model 2, the most influential diabetes-specific factors were their baseline diabetes spending (relative influence: 63.9), average adherence to oral diabetes medications (25.6), and number of unique diabetes medications (4.5). In Model 3, the most influential potentially-modifiable factors for that same group were their average adherence to medications (relative influence: 39.9), number of office visits (20.1), average adherence to oral diabetes medications (15.3), and number of Emergency Room (ER) visits (6.1).

### Sensitivity analyses: 40% threshold to define low-cost spending in the baseline year

We repeated the trajectory modeling and prediction modeling using a 40% threshold, which resulted in 15,017 patients (median baseline diabetes spending: $1255). The best-fitting trajectory model (Appendix Figure 6) indicated similar groupings and spending patterns across 5 groups as in the 90% threshold cohort, such as a minimal-user group (15.2%), a low-cost group (30.2%), a rising-cost group (5.7%), a moderate-cost group (28.4%), and a high-cost group (21.3%). Corresponding C-statistics for these groups are shown in Appendix Table 4. The results were largely similar as with the 90% threshold, although predictive ability was slightly lower owing largely to the smaller sample size.

## Discussion

Using data-driven approaches, we identified distinct diabetes spending patterns among a nationally-representative cohort of Medicare patients with type 2 diabetes who were initially low utilizers, including a definable group of patients whose costs began to rise progressively late in the first year of follow-up. These patterns could be predicted using baseline characteristics, including diabetes-specific factors and factors that may be potentially modifiable.

Current efforts to predict healthcare spending largely focus on predicting a composite value, such as total yearly diabetes spending, or a threshold-based measure, like being in the top 5% of spending, both of which collapse spending into a single static value [6, 9, 12, 40, 41]. Two recently-published approaches offer other cluster-based solutions to elucidate patterns of spending [40, 42]. For instance, researchers recently identified patients with initially low spending levels whose costs bloomed in the subsequent year using a threshold-based approach [17]. To our knowledge, neither of these approaches have been applied to patients with specific chronic conditions, such as type 2 diabetes.

Our findings support the conclusion that patients may have dynamic patterns of spending over longer periods of time that can be potentially meaningful, with implications on whom to outreach for intervention as well as when to do so [13, 14]. The ability to potentially discriminate between patients with differing diabetes spending patterns using variables measured at baseline could better target interventions to those who are at greatest need [41]. Many healthcare organizations, insurers, researchers, and policymakers make predictions and identify patients for cost-containment interventions using these types of administrative data [40, 41]. If successful, using these longer time horizons could allow for more time to implement potential cost-containment interventions for type 2 diabetes [41]. The ability to better leverage these routinely-collected data for predictions with more dynamic cost-modeling methods by chronic condition holds wide potential for possible interventions.

The findings of the most influential predictors also offer several noteworthy suggestions for potential diabetes-specific interventions in patients who previously had low spending levels. First, as observed in prior work, adherence to medication, both all medications and diabetes-specific medications, appears to be an important differentiator of patients in different groups, especially those with progressively rising costs; mean adherence is presented in this manuscript but adherence has been known to have meaningful underlying variations even if means are similar [43–46]. Adherence to medication has been shown in a number of contexts to contribute to the avoidance of poor health outcomes [44, 45, 47, 48]. Of note, while mean adherence appeared similar at baseline, it is known that there are important variations in adherence that composite metrics such as average adherence do not always represent, which could have explained why it was an important predictor, especially in interactions with other variables [28, 49]. Number of physician office visits and unique physicians may also be indicators of whether patients are getting sufficient care to prevent future escalation of diabetes problems [26]. Notably, non-modifiable diabetes factors, such as indicators of clinical progression like presence of neuropathy, nephropathy, or retinopathy, were not particularly influential in the boosted prediction models. Of course, one of the most influential predictors was baseline diabetes spending for several trajectory groups; thus, even though the groups had fairly similar spending in the baseline year (Appendix Figure 4), baseline spending is an important consideration when building prediction models and potentially targeting interventions. Insulin costs in particular could also be a key contributor to healthcare costs in these patients [1]. Together, these findings suggest that there are possible opportunities for the provision of interventions, such as adherence interventions or interventions that increase access to care, to prevent escalating complications and costs in diabetes. Future work should also explore how to apply these results in interventions and how these results replicate in other population, including electronic tools to build these prediction models.

There are several limitations. First, we examined trajectories from January to December; patients with incomplete enrollment or other policy start and end dates may have different spending patterns. The variables included in the prediction models may also not be exhaustive, and although we used validated algorithms for these variables where possible, they may not be sufficiently sensitive. While we used the 90% threshold from prior work to identify low spenders at baseline, the beneficiaries who were classified in the “high-cost” trajectory may also have had elevated spending to start. Trajectory modeling also provides predicted group membership within a cluster; while beneficiaries were assigned to their closest cluster, there could be some within-group heterogeneity. Given the nature of the data, we also do not have information about patients’ glycemic control at baseline, although this would apply to others using these data as well. These results may also not generalize to non-Medicare Fee-For-Service beneficiaries or younger adults, and the data are from several years ago (owing, in part, to an administrative lag in Medicare data). However, given that costs for diabetes are only continuing to increase and rates of type 2 diabetes are growing progressively in younger populations, we expect that these findings will continue to remain relevant [1]. Finally, some misclassification of the cohort is possible due to the nature of claims data; however, we used validated algorithms to define diabetes and other comorbidities to the extent possible.

## Conclusion

Many healthcare organizations use claims data to identify and predict patients for cost-containment interventions for people living with diabetes. The approach we describe could help inform the design and timing of cost-containment interventions, such as medication adherence or interventions that enhance access to care, and target them to those at greatest need in patients with type 2 diabetes.

## Supplementary information

**Additional file 1: Appendix Figure 1.** Study Design. **Appendix Table 1.** List of medications for diabetes. **Appendix Figure 2.** Percentiles of diabetes-specific spending in the baseline year. **Appendix Table 2.** Patient eligibility criteria. **Appendix Figure 3.** Trajectory modeling of two-year diabetes-specific spending using other numbers of groups. **Appendix Table 3.** Predicted probabilities for each trajectory group. **Appendix Figure 4.** Baseline monthly mean diabetes spending by trajectory group assignment spanning the baseline year and two follow-up years. **Appendix Figure 5.** Relative influence of variables for predicting group membership for models including diabetes-specific and potentially-modifiable predictors (all predictors shown). **Appendix Figure 6.** Two-year diabetes spending patterns using trajectory modeling: 40% cutpoint for determining low spending levels at baseline. **Appendix Table 4.** Ability of models to predict two-year diabetes spending trajectory groups: 40% cutpoint

## Data Availability

The data that support the findings of this study are available from the Research Data Assistance Center (ResDAC) from the Centers for Medicare and Medicaid Services, but restrictions apply to the availability of these data, which were used under license for the current study, and so are not publicly available. Data are however available from the authors upon reasonable request and with permission of ResDAC.

## Abbreviations

BIC: Bayesian information criterion
COPD: Chronic Obstructive Pulmonary Disease
ER: Emergency Room
ICD-9: International Classification of Diseases, 9th edition
GLP-1: Glucagon-like peptide 1
HS: High School
IRB: Institutional Review Board
MI: Myocardial Infarction
NIH: National Institutes of Health
NIHCM: National Institute for Health Care Management
PDC: Proportion of Days Covered
ResDAC: Research Data Assistance Center
SD: Standard Deviation
STROBE: Strengthening the Reporting of Observational Studies in Epidemiology

## References

[1] American Diabetes A (2018). Economic costs of diabetes in the U.S. in 2017. Diabetes Care.

[2] Hasche J, Ward C, Schluterman N. Diabetes occurrence, costs, and access to care among medicare beneficiaries aged 65 years and over*.* Centers for Medicare and Medicaid Services Office of Enterprise Data & Analytics; 2017.

[3] Garber AJ, Abrahamson MJ, Barzilay JI (2018). Consensus statement by the American Association of Clinical Endocrinologists and American College of endocrinology on the comprehensive type 2 diabetes management algorithm - 2018 executive summary. Endocr Pract.

[4] Huang ES, Liu JY, Moffet HH, John PM, Karter AJ (2011). Glycemic control, complications, and death in older diabetic patients: the diabetes and aging study. Diabetes Care.

[5] Qaseem A, Wilt TJ, Kansagara D (2018). Hemoglobin A1c targets for glycemic control with pharmacologic therapy for nonpregnant adults with type 2 diabetes mellitus: a guidance statement update from the American College of Physicians. Ann Intern Med.

[6] Meyers JL, Parasuraman S, Bell KF, Graham JP, Candrilli SD (2014). The high-cost, type 2 diabetes mellitus patient: an analysis of managed care administrative data. Arch Public Health.

[7] Sales AE, Liu CF, Sloan KL (2003). Predicting costs of care using a pharmacy-based measure risk adjustment in a veteran population. Med Care.

[8] Fishman PA, Goodman MJ, Hornbrook MC, Meenan RT, Bachman DJ, O'Keeffe Rosetti MC (2003). Risk adjustment using automated ambulatory pharmacy data: the RxRisk model. Med Care.

[9] Powers CA, Meyer CM, Roebuck MC, Vaziri B (2005). Predictive modeling of total healthcare costs using pharmacy claims data: a comparison of alternative econometric cost modeling techniques. Med Care.

[10] Forrest CB, Lemke KW, Bodycombe DP, Weiner JP (2009). Medication, diagnostic, and cost information as predictors of high-risk patients in need of care management. Am J Manag Care.

[11] Yarger S, Rascati K, Lawson K, Barner J, Leslie R (2008). Analysis of predictive value of four risk models in Medicaid recipients with chronic obstructive pulmonary disease in Texas. Clin Ther.

[12] Mihaylova B, Briggs A, O'Hagan A, Thompson SG (2011). Review of statistical methods for analysing healthcare resources and costs. Health Econ.

[13] Martin AB, Hartman M, Washington B, Catlin A (2017). National Health Expenditure Accounts T. National Health Spending: faster growth in 2015 as coverage expands and utilization increases. Health Aff.

[14] Druss BG, Marcus SC, Olfson M, Tanielian T, Elinson L, Pincus HA (2001). Comparing the national economic burden of five chronic conditions. Health Aff.

[15] Ziaeian B, Fonarow GC (2016). The prevention of hospital readmissions in heart failure. Prog Cardiovasc Dis.

[16] Barnett ML, Hsu J, McWilliams JM (2015). Patient characteristics and differences in hospital readmission rates. JAMA Intern Med.

[17] Tamang S, Milstein A, Sorensen HT (2017). Predicting patient ‘cost blooms’ in Denmark: a longitudinal population-based study. BMJ Open.

[18] Lauffenburger JC, Franklin JM, Krumme AA (2017). Longitudinal patterns of spending enhance the ability to predict costly patients: a novel approach to identify patients for cost containment. Med Care.

[19] Krumme AA, Glynn RJ, Schneeweiss S (2018). Medication synchronization programs improve adherence to cardiovascular medications and health care use. Health Aff.

[20] Khokhar B, Jette N, Metcalfe A (2016). Systematic review of validated case definitions for diabetes in ICD-9-coded and ICD-10-coded data in adult populations. BMJ Open.

[21] Austin PC, Ghali WA, Tu JV (2003). A comparison of several regression models for analysing cost of CABG surgery. Stat Med.

[22] Nuckols TK, Escarce JJ, Asch SM (2013). The effects of quality of care on costs: a conceptual framework. Milbank Q.

[23] Benner JS, Glynn RJ, Mogun H, Neumann PJ, Weinstein MC, Avorn J (2002). Long-term persistence in use of statin therapy in elderly patients. JAMA..

[24] Choudhry NK, Shrank WH, Levin RL (2009). Measuring concurrent adherence to multiple related medications. Am J Manag Care.

[25] Stolpe S, Kroes MA, Webb N, Wisniewski T (2016). A systematic review of insulin adherence measures in patients with diabetes. J Manag Care Spec Pharm.

[26] Goetzel RZ, Pei X, Tabrizi MJ (2012). Ten modifiable health risk factors are linked to more than one-fifth of employer-employee health care spending. Health Aff.

[27] Yusuf S, Hawken S, Ounpuu S (2004). Effect of potentially modifiable risk factors associated with myocardial infarction in 52 countries (the INTERHEART study): case-control study. Lancet..

[28] Franklin JM, Shrank WH, Pakes J (2013). Group-based trajectory models: a new approach to classifying and predicting long-term medication adherence. Med Care.

[29] Jones BL, Nagin DS (2007). Advances in group-based trajectory modeling and a SAS procedure for estimating them. Sociol Methods Res.

[30] Jones BL, Nagin DS, Roeder K (2001). A SAS procedure based on mixture models for estimating developmental trajectories. Sociol Methods Res.

[31] Li Y, Zhou H, Cai B (2014). Group-based trajectory modeling to assess adherence to biologics among patients with psoriasis. Clinicoecon Outcomes Res.

[32] Franklin JM, Krumme AA, Tong AY, et al. Association between trajectories of statin adherence and subsequent cardiovascular events. Pharmacoepidemiol Drug Saf. 2015;24:1105–13. 10.1002/pds.3787 25903307

[33] Franklin JM, Shrank WH, Lii J (2015). Observing versus predicting: initial patterns of filling predict long-term adherence more accurately than high-dimensional modeling techniques. Health Serv Res.

[34] Koh HC, Tan G (2005). Data mining applications in healthcare. J Healthc Inf Manag.

[35] Robinson JW (2008). Regression tree boosting to adjust health care cost predictions for diagnostic mix. Health Serv Res.

[36] Varian HR (2014). Big data: new tricks for econometrics. J Econ Perspect.

[37] Waljee AK, Higgins PD, Singal AG (2014). A primer on predictive models. Clin Transl Gastroenterol.

[38] Steyerberg EW, Vickers AJ, Cook NR (2010). Assessing the performance of prediction models: a framework for traditional and novel measures. Epidemiology..

[39] Cook NR (2007). Use and misuse of the receiver operating characteristic curve in risk prediction. Circulation..

[40] Powers BW, Yan J, Zhu J (2019). Subgroups of high-cost medicare advantage patients: an observational study. J Gen Intern Med.

[41] Stadhouders N, Kruse F, Tanke M, Koolman X, Jeurissen P (2019). Effective healthcare cost-containment policies: a systematic review. Health Policy.

[42] Yan J, Linn KA, Powers BW (2019). Applying machine learning algorithms to segment high-cost patient populations. J Gen Intern Med.

[43] Choudhry NK, Glynn RJ, Avorn J (2014). Untangling the relationship between medication adherence and post-myocardial infarction outcomes: medication adherence and clinical outcomes. Am Heart J.

[44] Adams AS, Trinacty CM, Zhang F (2008). Medication adherence and racial differences in A1C control. Diabetes Care.

[45] Iuga AO, McGuire MJ (2014). Adherence and health care costs. Risk Manag Healthc Policy.

[46] Whelton PK, Carey RM, Aronow WS (2018). 2017 ACC/AHA/AAPA/ABC/ACPM/AGS/APhA/ASH/ASPC/NMA/PCNA guideline for the prevention, detection, evaluation, and management of high blood pressure in adults: a report of the American College of Cardiology/American Heart Association Task Force on Clinical Practice Guidelines. J Am Coll Cardiol.

[47] Cutrona SL, Choudhry NK, Fischer MA (2010). Modes of delivery for interventions to improve cardiovascular medication adherence. Am J Manag Care.

[48] Cutrona SL, Choudhry NK, Fischer MA (2012). Targeting cardiovascular medication adherence interventions. J Am Pharm Assoc.

[49] Franklin JM, Krumme AA, Shrank WH, Matlin OS, Brennan TA, Choudhry NK (2015). Predicting adherence trajectory using initial patterns of medication filling. Am J Manag Care.

