# Supplementary material for: Not there yet: using data-driven methods to predict who becomes costly among low-cost patients with type 2 diabetes
Source: BMC Endocr Disord. 2020 Aug 17;20:125. doi: 10.1186/s12902-020-00609-1 (PMC7433196; doi:10.1186/s12902-020-00609-1)
Supplement: Supplementary file 1 — Additional file 1: Appendix Figure 1. Study Design. Appendix Table 1. List of medications for diabetes. Appendix Figure 2. Percentiles of diabetes-specific spending in the baseline year. Appendix Table 2. Patient eligibility criteria. Appendix Figure 3. Trajectory modeling of two-year diabetes-specific spending using other numbers of groups. Appendix Table 3. Predicted probabilities for each trajectory group. Appendix Figure 4. Baseline monthly mean diabetes spending by trajectory group assignment spanning the baseline year and two follow-up years. Appendix Figure 5. Relative influence of variables for predicting group membership for models including diabetes-specific and potentially-modifiable predictors (all predictors shown). Appendix Figure 6. Two-year diabetes spending patterns using trajectory modeling: 40% cutpoint for determining low spending levels at baseline. Appendix Table 4. Ability of models to predict two-year diabetes spending trajectory groups: 40% cutpoint [file 12902_2020_609_MOESM1_ESM.docx]

**APPENDIX**

**Appendix Figure 1.** Study Design

Cohort entry date 1/1/2012

1/1/2011

12/31/2013

Two-year diabetes spending outcomes

Baseline predictors

1/1/2013

**Appendix Table 1.** List of medications for diabetes

| **Medications (by generic name)** |
| --- |
| **Oral glucose-lowering agents**   - Acarbose - Acetohexamide - Alogliptin - Canagliflozin - Chlorpropamide - Dapagliflozin - Empagliflozin - Glimepiride - Glipizide - Glyburide - Linagliptin - Metformin - Miglitol - Nateglinide - Pioglitazone - Repaglinide - Rosiglitazone - Saxagliptin - Sitagliptin - Tolazamide - Tolbutamide - Troglitazone   **Insulins**   - Insulin aspart - Insulin detemir - Insulin glargine - Insulin isophane - Insulin lente - Insulin lispro protamine - Insulin lispro - Insulin NPH - Insulin regular - Insulin ultralente   **Non-insulin injectables**   - Exenatide - Liraglutide - Albiglutide - Pramlintide acetate - Dulaglutide |

**Appendix Figure 2.** Percentiles of diabetes-specific spending in the baseline year

**Annual costs in US$**

**Percentile (1 to 100)**


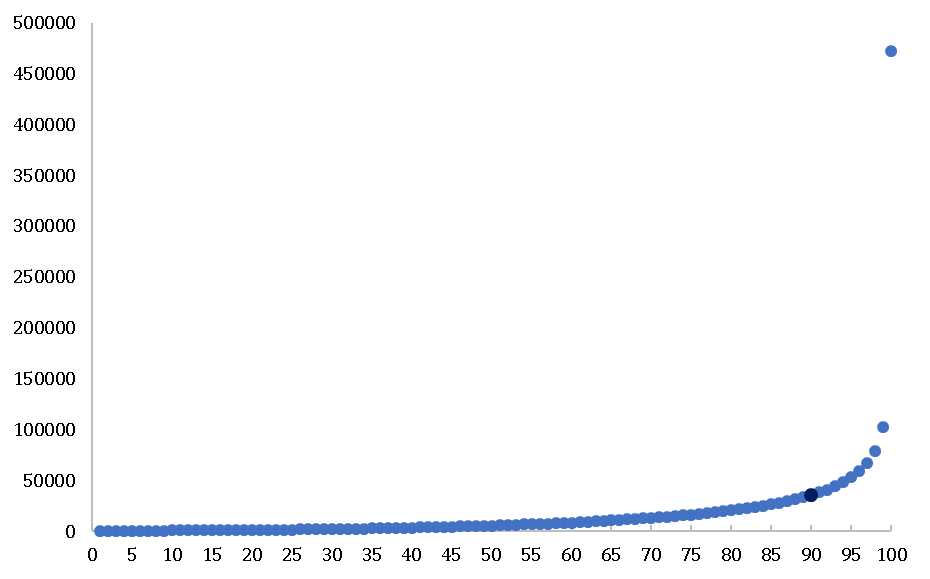


**Appendix Table 2.** Patient eligibility criteria

| Criterion | | N |
| --- | --- | --- |
| Enrolled on 1/1/12 in medical and pharmacy benefits | | 550,215 |
| Age ≥65 years on 1/1/11 | | 433,561 |
| Continuous enrollment from 1/1/11 to 12/31/13 | | 329,476 |
| Type 2 diabetes diagnosis | | 37,544 |
| Bottom 90^th^ percentile | | 33,789 |
|  |  |  |

**Appendix Figure 3.** Trajectory modeling of two-year diabetes-specific spending using other numbers of groups

1. **Two-group model**

BIC: 2382993


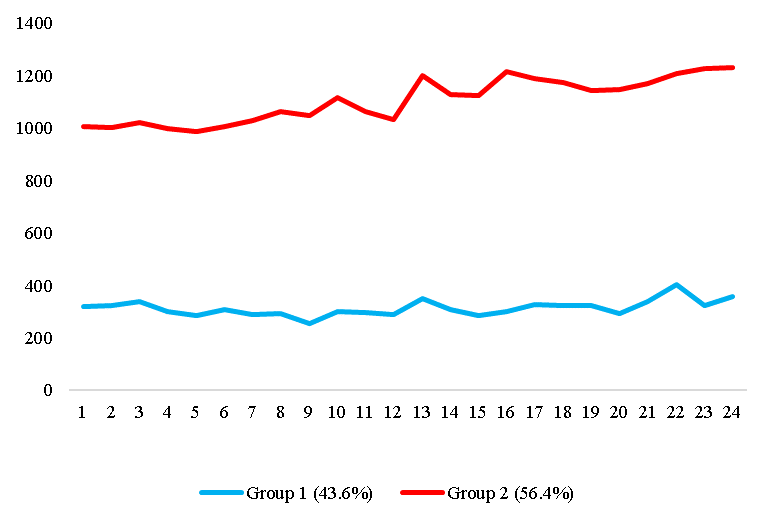


**Monthly costs (in US $)**

**Months**

1. **Three-group model**

**Monthly costs (in US $)**

BIC: 2364511


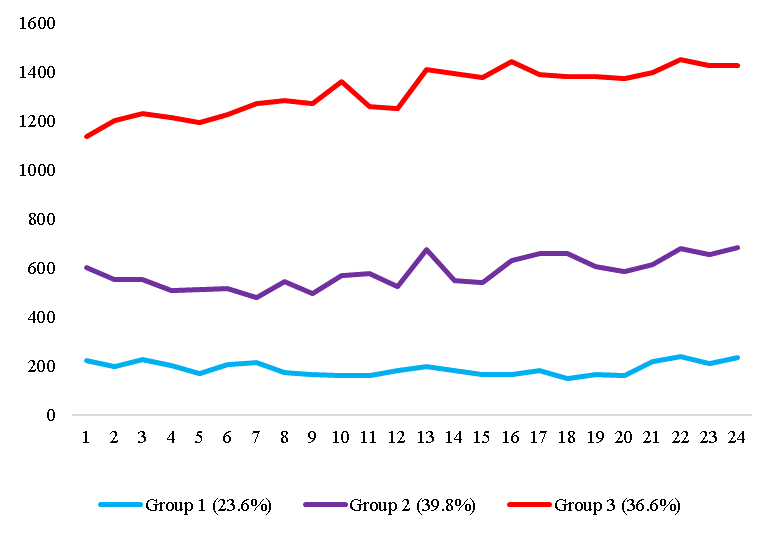


**Months**

1. **Four-group model**

BIC: 2360008


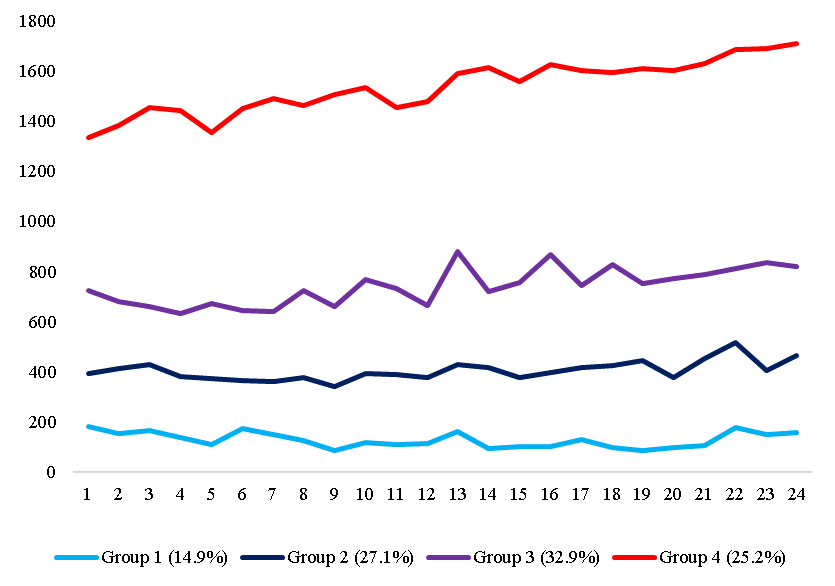


**Months**

**Monthly costs (in US $)**

**Months**

1. **Six-group model**

BIC: 2357060


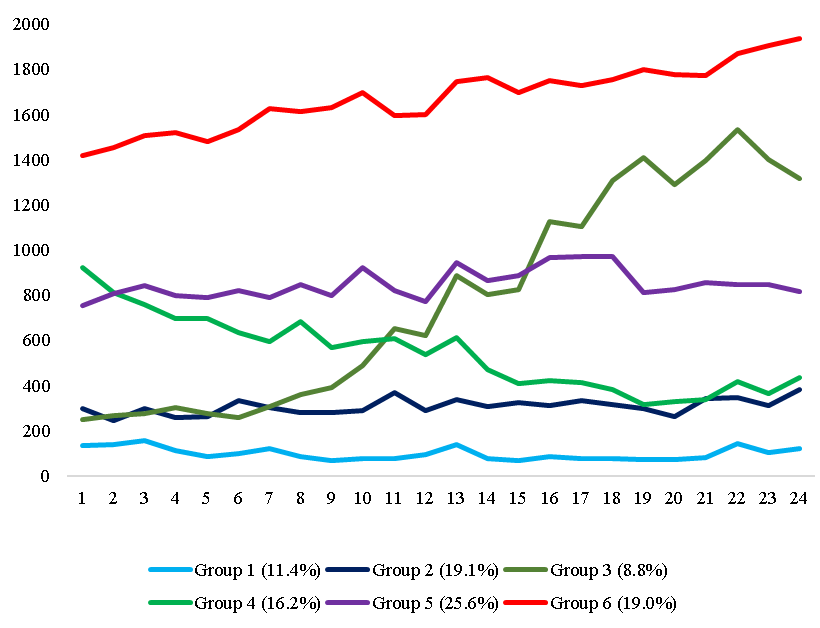


**Months**

**Monthly costs (in US $)**

**Appendix Table 3.** Predicted probabilities for each trajectory group

| **Trajectory group** | **Mean (SD) predicted probability of trajectory group membership** | **% of patients with >0.70 membership probability** |
| --- | --- | --- |
| Group 1: Minimal-user | 0.92 (0.13) | 89.2% |
| Group 2: Low-cost | 0.86 (0.15) | 83.5% |
| Group 3: Rising-cost | 0.76 (0.18) | 62.2% |
| Group 4: Moderate-cost | 0.83 (0.15) | 76.5% |
| Group 5: High-cost | 0.92 (0.13) | 89.8% |

**Appendix Figure 4.** Baseline monthly mean diabetes spending by trajectory group assignment spanning the baseline year and two follow-up years

**Monthly costs (in US $)**

**Appendix Figure 5.** Relative influence of variables for predicting group membership for models including diabetes-specific and potentially-modifiable predictors (all predictors shown)

1. **Diabetes-specific predictors**

**Relative influence**

1. **Potentially-modifiable predictors**

**Relative influence**

**Appendix Figure 6.** Two-year diabetes spending patterns using trajectory modeling: 40% cutpoint for determining low spending levels at baseline

**Months**

**Monthly costs (in US $)**

**Appendix Table 4.** Ability of models to predict two-year diabetes spending trajectory groups: 40% cutpoint

|  | **Validated C-statistics** | | |
| --- | --- | --- | --- |
| **Group** | **Model 1:**  **All baseline predictors** | **Model 2: Diabetes predictors** | **Model 3: Potentially-modifiable predictors** |
| Group 1: Minimal user | 0.824 | 0.801 | 0.786 |
| Group 2: Low-cost | 0.734 | 0.703 | 0.698 |
| Group 3: Rising-cost | 0.625 | 0.616 | 0.611 |
| Group 4: Moderate-cost | 0.645 | 0.625 | 0.618 |
| Group 5: High-cost | 0.839 | 0.821 | 0.813 |
